# Perioperative complication incidence and risk factors for retroperitoneal neuroblastoma in children: analysis of 571 patients

**DOI:** 10.1007/s12519-023-00773-z

**Published:** 2023-12-09

**Authors:** Min He, Jia-Bin Cai, Xuan Wu, Yin-Bing Tang, Jin-Yan Wang, Jun-Qin Mao, Ji-Jun Chen, Li-Feng Zhang, Zhong-Hai Guan, Jie-Ni Xiong, Wan-Xin Peng, Jin-Hu Wang, Ting Tao

**Affiliations:** 1 Pediatric Cancer Research Center, National Clinical Research Center for Child Health, Hangzhou, China; 2 Department of Surgical Oncology, Children’s Hospital, Zhejiang University School of Medicine, Hangzhou, China; 3 Cancer Center, Zhejiang University, No. 3333 Binsheng Rode, Hangzhou, China

**Keywords:** Complication, Neuroblastoma, Risk factors, Surgery

## Abstract

**Background:**

Surgery plays an important role in the treatment of neuroblastoma. Perioperative complications may impact the course of neuroblastoma treatment. To date, comprehensive analyses of complications and risk factors have been lacking.

**Methods:**

Patients with retroperitoneal neuroblastoma undergoing tumor resection were retrospectively analyzed between 2014 and 2021. The data collected included clinical characteristics, operative details, operative complications and postoperative outcomes. Risk factors for perioperative complications of retroperitoneal neuroblastoma were analyzed.

**Results:**

A total of 571 patients were enrolled in this study. Perioperative complications were observed in 255 (44.7%) patients. Lymphatic leakage (28.4%), diarrhea (13.5%), and injury (vascular, nerve and organ; 7.5%) were the most frequent complications. There were three operation-related deaths (0.53%): massive hemorrhage (*n* = 1), biliary tract perforation (*n* = 1) and intestinal necrosis (*n* = 1). The presence of image-defined risk factors (IDRFs) [odds ratio (OR) = 2.09, *P* < 0.01], high stage of the International Neuroblastoma Risk Group staging system (INRGSS) (OR = 0.454, *P* = 0.04), retroperitoneal lymph node metastasis (OR = 2.433, *P* = 0.026), superior mesenteric artery encasement (OR = 3.346, *P* = 0.003), and inferior mesenteric artery encasement (OR = 2.218, *P* = 0.019) were identified as independent risk factors for perioperative complications.

**Conclusions:**

Despite the high incidence of perioperative complications, the associated mortality rate was quite low. Perioperative complications of retroperitoneal neuroblastoma were associated with IDRFs, INRGSS, retroperitoneal lymph node metastasis and vascular encasement. Patients with high-risk factors should receive more serious attention during surgery but should not discourage the determination to pursue total resection of neuroblastoma.

Video Abstract (MP4 94289 KB)

**Supplementary Information:**

The online version contains supplementary material available at 10.1007/s12519-023-00773-z.

## Introduction

Neuroblastoma (NB) is the most common extracranial solid tumor in childhood and is responsible for 15% of all pediatric oncology deaths [1]. High-risk NB still has a poor prognosis, with a 50%–60% 5-year event-free survival rate [2]. A report from the International Society of Pediatric Oncology Europe Neuroblastoma Group High-Risk Neuroblastoma 1 study showed improved event-free and overall survival associated with complete macroscopic excision [3]. NB most commonly develops in the posterior peritoneal region, and the adrenal gland is the most frequent origin [4]. Complete resection of retroperitoneal NB is clearly difficult to achieve when extensive lymph node metastasis and vascular encasement are present. Since Kiely first came up with the technique of dissection in the subadventitial plane, the total resection rate of tumors has been remarkably improved [5, 6].

Pediatric surgeons strive for gross total resection (GTR), but there is a scarcity of data with respect to complications associated with operations for retroperitoneal NB. According to the reports that have been published thus far, lymphatic leakage (LL) and diarrhea are common complications after NB resection, and major vascular injury may lead to serious consequences [7, 8]. The presence of image-defined risk factors (IDRFs) at resection was associated with a greater incidence of intraoperative complications [9, 10]. However, large sample sizes, comprehensive analyses of all complications and risk factor assessments were absent from these studies.

The aim of this study was to determine the incidence of all perioperative complications in patients undergoing retroperitoneal NB resection and identify risk factors associated with significant complications.

## Methods

### Patients

**Fig. 1:**
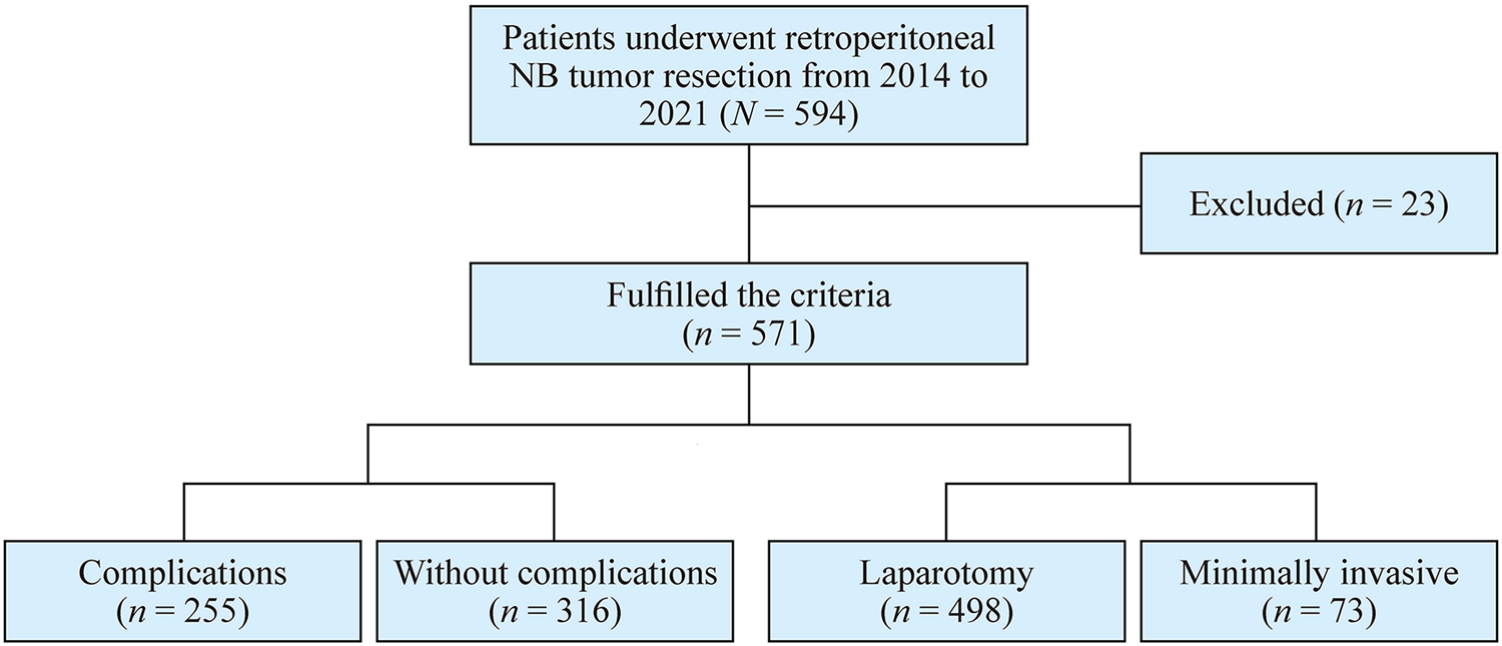
Flowchart of this study. *NB* neuroblastoma

A total of 594 patients underwent retroperitoneal NB tumor resection from January 2014 to December 2021 in our center, of whom 571 were enrolled in this study and fulfilled the following eligibility criteria: (1) primary retroperitoneal NB; and (2) tumor resection was performed instead of tumor biopsy. Twenty-three cases were excluded due to dysfunction of vital organs and major illnesses such as heart or brain disease (Fig. 1). Informed consent to participate in the study was obtained from participants (or their parent or legal guardian in the case of children under 16).

All patients underwent enhanced computed tomography and vascular three-dimensional reconstruction to determine the presence of IDRFs before the operation. NB was diagnosed in either of the following ways: (1) unequivocal pathologic diagnosis by tumor resection or biopsy; and (2) bone marrow aspirate or trephine biopsy contains unequivocal tumor cells.

### Surgery

Localized NB in stage L1 could be operated on by a minimally invasive approach, while laparotomy surgery was recommended for tumors with IDRFs. Chemotherapy should be administered to lower the IDRFs if it is present before surgery. Tumor excision was attempted after 2–4 cycles of chemotherapy if necessary, depending on the response. All procedures were performed by the same group of surgeons, all of whom were senior pediatric oncology surgeons with more than 10 years of practice. The detailed protocol, including inductive chemotherapy, surgery and consolidation therapy, of each International Neuroblastoma Risk Group (INRG) was based on the CCCG-NB-2015 Regimen [11].

Removal of all macroscopic tumors, including metastatic lymph nodes and suspicious lesions on imaging, was defined as GTR, and incomplete tumor removal (residual macroscopic tumor, tumor removal less than 90%) was defined as partial resection (PR). The basic principles of surgical resection include the accurate and safe display of vital vascular anatomy with optimal exposure of the tumor to achieve GTR as much as possible while avoiding unnecessary organ removal.

### Outcomes

The primary outcome was the occurrence of direct, common and major complications, including hemorrhage (20 mL/kg or more), vascular injury, unplanned organ resection, secondary hypertension, LL, diarrhea, important structural injury, and any organ failure. The secondary outcome measured included individual complications (minor complications), including wound complications, abdominal infection, intestinal obstruction, and hypoglycemia.

LL was defined as (1) the milky-white drain fluid lasted up to 48 hours; (2) the drain output was more than 100 mL/day and did not decrease 48 hours after the operation; and (3) the triglyceride of the drainage fluid was greater than 1.13 mmol/L, and meeting any of the criteria, the diagnosis could be made.

Diarrhea was defined as three or more loose stools per day lasting for more than 3 days. Intestinal obstruction was considered a minor complication in this cohort, as no patients underwent surgery due to postoperative intestinal obstruction; nevertheless, all of these individuals improved with conservative care and did not suffer significantly.

### Risk factors

Age, sex, maximum tumor diameter at operation, location, tumor origin, Shimada classification, IDRFs, INRG stage, INRG risk group, MYCN status, retroperitoneal lymph node metastasis, operation style, operation time, surgical excision and avascular encasement were evaluated as potential risk factors for complications. Vascular encasement in this study was defined as having tumors wrapping more than 75% of the surrounding blood vessels, including the aorta, celiac trunk, superior mesenteric artery, renal artery, and inferior mesenteric artery.

### Statistical analysis

For statistical analysis, IBM SPSS software (Version 26.0.0; Armonk, NY, IBM Corp.) was used. The relationship between patient characteristics and perioperative complications was evaluated using Chi-squared test. Univariable analysis produced significant variables, and risk factors related to postoperative complications were finally filtered out by multivariable conditional logistic regression. A probability of type I error of 5% (*P* < 0.05) was used to determine statistical significance.

## Results

### Patients

The key characteristics of the patients who were eligible for the criteria are listed in Table 1.

**Table 1:** Relationship between patient characteristics and complications

Characteristic	Complications		*χ*^*2*^	*P*
	Present	Absent		
No. of patients, *n* (%)	337 (59)	234 (41)		
Sex				
Male	115	173	0.265	0.607
Female	119	164		
Age (mon)				
≤ 18	54	86	0.445	0.505
> 18	180	251		
IDRFs				
No	7	137	167.151	< 0.001
Yes	227	200		
INRGSS stage				
L1	6	101	90.571	< 0.001
L2	66	61		
M	162	167		
Ms	0	8		
NMYC status				
Not amplified	133	260	18.756	< 0.001
Amplified	68	54		
Missing	33	23		
Shimada type				
NB	192	223	28.890	< 0.001
GNB intermixed	21	51		
GN maturing	3	39		
GNB nodular	18	24		
Location				
Left	123	160	2.195	0.790
Right	99	159		
Bilateral	12	18		
Tumor origin				
Adrenal	215	283	8.118	0.007
SC	19	54		
RLNM				
No	34	182	91.510	< 0.001
Yes	200	155		
MTD at operation (cm)				
≤ 5	91	187	15.234	< 0.001
> 5	143	150		
INRG risk group				
Very low	11	95	61.165	< 0.001
Low	26	55		
Intermediate	35	28		
High	162	159		
Operation style				
Laparotomy	229	269	40.315	< 0.001
MI	5	68		
Operation time (h)				
< 3	14	89	104.315	< 0.001
3–5	67	167		
> 5	153	81		
Vascular encasement				
No	15	201	166.407	< 0.001
Yes	219	136		
Aorta encasement				
No	31	250	205.176	< 0.001
Yes	203	87		
CT encasement				
No	70	292	191.553	< 0.001
Yes	164	45		
SMA encasement				
No	63	294	214.429	< 0.001
Yes	171	43		
RA encasement				
No	61	226	92.837	< 0.001
Yes	173	111		
IMA encasement				
No	141	316	97.066	< 0.001
Yes	93	21		
Surgical excision				
GTR	207	316	5.052	0.025
PR	27	21		

*IDRF* image-defined risk factors, *INRGSS* The International Neuroblastoma Risk Group staging system, *NB* neuroblastoma, *GNB* ganglioneuroblastoma, *GN* ganglioneuroma, *SC* sympathetic chain, *RLNM* retroperitoneal lymph node metastasis, *MTD* maximum tumor diameter, *INRG* International Neuroblastoma Risk Group, *MI* minimally invasive, *CT* celiac trunk, *SMA* superior mesenteric artery, *RA* renal artery, *IMA* inferior mesenteric artery, *GTR* gross total resection, *PR* partial resection

### Operative factors

Laparotomy was performed in 498 patients and minimally invasive in 73 patients, including laparoscopic surgery in 59 patients and DaVinci robotic surgery in 14 patients. The tumors in 524 cases were GTR, while 49 cases were PR.

### Perioperative complications

Perioperative complications were observed in 255 (44.7%) of 571 patients. Primary complications occurred in 234 (41.0%) patients, while minor complications occurred in 31 (5.4%). The incidence of intraoperative and postoperative complications was 6.8% and 39.6%, respectively. There were 3 (0.53%) operation-related deaths: massive hemorrhage (*n* = 1), biliary tract perforation (*n* = 1), and intestinal necrosis (*n* = 1).

LL (28.4%), diarrhea (13.5%), and injury (vascular, nerve and organ; 7.5%) were the most frequent complications. Vascular injury occurred in 19 (3.3%) patients, including the aorta, renal artery, superior mesenteric artery, inferior mesenteric artery, and splenic artery. All other complications (hypertension, unplanned organ resection, infection or minor complications) occurred in < 4.2% of patients. All complications and their incidences are illustrated in Table 2.

**Table 2:** Incidence of perioperative complications

Complications	*n* (%)
Lymphatic leakage	162 (28.4)
Diarrhea	77 (13.5)
Important structural injury	43 (7.5)
Vascular	19 (3.3)
Intestinal tract	7 (1.2)
Ureter	5 (0.8)
Pancreas	5 (0.8)
Common bile duct	4 (0.7)
Nerve	3 (0.5)
Hypertension	24 (4.2)
Massive hemorrhage	6 (1.1)
Unplanned organ resection	20 (3.5)
Minor complications	31 (5.4)
Hypoglycemia	11 (1.9)
Intestinal obstruction	7 (1.2)
Abdominal infection	7 (1.2)
Incision-related complications	6 (1.1)

### Risk factors

#### Univariable analysis

The relationship between factors and perioperative complications was analyzed (Table 1). Complications were more likely to be associated with larger tumor size, adrenal primary tumor, Shimada classification as NB, presence of IDRFs, high stage of INRG staging system (INRGSS), high-risk group of INRG, MYCN amplification, retroperitoneal lymph node metastasis, laparotomy surgery, long period of operations, GTR, and vascular encasement.

LL and diarrhea were more likely to be associated with larger tumor size, Shimada classification as NB, presence of IDRFs, high stage of INRGSS, high-risk group of INRG, MYCN amplification, retroperitoneal lymph node metastasis, laparotomy surgery, long period of operations, and vascular encasement (Table 3). There was PR in 6.2% in the absence of complications compared with 11.5% in their presence (*P* = 0.025).

**Table 3:** Relationship between patient characteristics with lymphatic leakage and diarrhea

Characteristics	Lymphatic leakage		*P*	Diarrhea		*P*
	Present	Absent		Present	Absent	
Sex						
Male	80	208	0.751	37	251	0.653
Female	82	201		40	243	
Age (mon)						
≤ 18	33	107	0.147	16	124	0.412
< 18	129	302		61	370	
IDRFs						
No	3	141	< 0.001	2	142	< 0.001
Yes	159	268		75	352	
INRGSS stage						
L1	1	106	< 0.001	3	104	< 0.001
L2	44	83		21	106	
M	117	212		53	276	
Ms	0	8		0	8	
NMYC status						
Not amplified	94	299	< 0.001	39	354	0.004
Amplified	52	70		24	98	
Missing	16	40		14	42	
Shimada type						
NB	133	282	< 0.001	55	360	< 0.001
GNB intermixed	15	57		12	60	
GN maturing	1	41		1	41	
GNB nodular	13	29		9	33	
Location						
Left	83	201	0.905	46	238	0.120
Right	72	185		29	228	
Bilateral	7	23		2	28	
Tumor origin						
Adrenal	149	349	0.052	72	426	0.228
SC	13	60		5	68	
RLNM						
No	17	199	< 0.001	11	205	< 0.001
Yes	145	210		66	289	
MTD at operation (cm)						
≤ 5	60	218	< 0.001	27	251	0.010
> 5	102	191		50	243	
INRG risk group						
Very low	5	101	< 0.001	5	101	0.004
Low	17	64		7	74	
Intermediate	21	42		13	50	
High	119	202		52	269	
Operation style						
Laparotomy	161	337	< 0.001	76	422	0.001
MI	1	72		1	72	
Operation time (h)						
< 3	6	97	< 0.001	6	97	< 0.001
3–5	47	187		17	217	
> 5	109	125		54	180	
Vascular encasement						
No	6	210	< 0.001	2	214	< 0.001
Yes	156	199		75	280	

*IDRF* image-defined risk factors, *INRGSS* The International Neuroblastoma Risk Group staging system, *NB* neuroblastoma, *GNB* ganglioneuroblastoma, *GN* ganglioneuroma, *SC* sympathetic chain, *RLNM* retroperitoneal lymph node metastasis, *MTD* maximum tumor diameter, *INRG* International Neuroblastoma Risk Group, *MI* minimally invasive

#### Multivariate analysis

The multivariate analyses of risk factors for perioperative complications are presented in Table 4. The presence of IDRFs [odds ratio (OR) = 2.09, 95% confidence interval (CI) = 1.387–3.15, *P* < 0.01], high stage of INRGSS (OR = 0.454, 95% CI = 0.213–0.968, *P* = 0.04), retroperitoneal lymph node metastasis (OR = 2.433, 95% CI = 1.115–5.31, *P* = 0.026), superior mesenteric artery encasement (OR = 3.346, 95% CI = 1.505–7.439, *P* = 0.003), and inferior mesenteric artery encasement (OR = 2.218, 95% CI = 1.14–4.312, *P* = 0.019) were identified as independent risk factors for perioperative complications.

**Table 4:** Multivariate analysis of risk factors for perioperative complications, lymphatic leakage, and diarrhea

Variables	Risk factors	*P*	OR	95% CI
Operative complications	IDRFs			
	Present	< 0.001	2.090	1.387–3.150
	INRGSS			
	Stage M	0.041	0.454	0.213–0.968
	RLNM			
	Present	0.026	2.433	1.115–5.310
	SMA encasement			
	Present	0.003	3.346	1.505–7.439
	IMA encasement			
	Present	0.019	2.218	1.140–4.312
Lymphatic leakage	RLNM			
	Present	0.030	2.580	1.098–6.062
	SMA encasement			
	Present	0.001	3.955	1.759–8.889
	IMA encasement			
	Present	0.040	0.531	0.288–0.978
Diarrhea	IMA encasement			
	Present	0.001	22.435	9.340–53.889

*IDRF* image-defined risk factors, *INRGSS* The International Neuroblastoma Risk Group staging system, *RLNM* retroperitoneal lymph node metastasis, *SMA* superior mesenteric artery, *IMA* inferior mesenteric artery, *OR* odds ratio, *CI* confidence interval

Retroperitoneal lymph node metastasis, superior mesenteric artery encasement, and inferior mesenteric artery encasement were identified as independent risk factors for LL (Table 4). Inferior mesenteric artery encasement was identified as an independent risk factor for diarrhea (Table 4).

#### Survival analysis

Five-year event-free survival (5-year EFS; 0.750 ± 0.027) and 5-year overall survival (5-year OS; 0.820 ± 0.025) were significantly higher without complications than with complications (5-year EFS: 0.523 ± 0.043; 5-year OS: 0.682 ± 0.044; *P* < 0.001; Table 5).

**Table 5:** Relationship between event-free survival, overall survival, and perioperative complication

Complication	No. of patients	EFS			OS		
		Events	5-year EFS ± SE	*P*	Events	5-year OS ± SE	*P*
Absent	337	70	0.750 ± 0.027	< 0.001	45	0.820 ± 0.025	< 0.001
Present	234	81	0.523 ± 0.043		50	0.682 ± 0.044	

*EFS* event-free survival, *OS* overall survival, *SE* standard error

## Discussion

In the present study, we analyzed the incidence of all perioperative complications and risk factors for perioperative complications in patients who underwent tumor resection for NB. IDRFs, high stage of INRGSS, retroperitoneal lymph node metastasis, and vascular encasement were significant risk factors for perioperative complications, yet MYCN amplification and tumor size were not significant risk factors. Furthermore, despite the high incidence of perioperative complications, the associated mortality rate was quite low.

Surgery plays an important role in the treatment of NB and is associated with improved survival [3, 12]. Compared with tumor PR, patients who underwent GTR had significantly decreased mortality at 3 years and 5 years [13]. However, there are conflicting viewpoints. Gehad reported that the extent of tumor resection had no impact on EFS and OS, and the concept of accepting incomplete resection to avoid serious complications was successful [14].

Theoretically, the incidence of complications increased with the extent of tumor resection. However, this was not the case in the present study. In fact, the surgeon was forced to terminate the procedure due to severe complications during the operation, such as vascular injury and bleeding. As a result, GTR is not achievable, which increases the incidence of complications in patients with PR. The reported incidence of complications of NB ranged from 20% to 50%, depending on sample size, surgeon’s experience, and the definition criteria for complications. In this study, the overall complication rate was 44.7%. LL, diarrhea, and injury were the most frequent complications in the cohort.

As of yet, there is no official definition of LL, and each study differs in its definition. The occurrence of LL was closely related to the scope of lymph node dissection, and skeletonization resection around the superior mesenteric artery was identified as a significant risk factor. Prophylactic mesenteric lymphatic ligation contributed to its effectiveness in the prevention of chylous fistulae [15]. In our experience, another way to prevent LL was to suture the side peritoneum in a meticulous manner so that the retroperitoneal area was completely isolated from the abdominal cavity. The side peritoneum was often removed along with the tumor. In that case, the mesocolon could be sutured to the liver and lateral abdominal wall on the right side, while the stomach and descending colon could be sutured to the diaphragm and lateral abdominal wall on the left side to close the posterior peritoneum. When LL occurred, encapsulated effusion formed in the enclosed space behind the peritoneum and no longer increased once the tension reached a certain level.

Diarrhea, one of the paraneoplastic syndromes, is due to hypersecretion of vasoactive intestinal peptide by the tumor and will disappear after tumor resection [16]. In contrast, postoperative diarrhea has rarely been reported. In fact, diarrhea was fairly routine after retroperitoneal NB resection, occurring in up to 13.5% of our cohort, and was significantly related to tumor dissection around the aorta and inferior mesenteric artery. It was inferred that the accompanying excision of sympathetic nerve fibers leading to autonomous nerve dysfunction might be the culprit. This type of diarrhea was characterized by being refractory to medication and having a long duration. The symptoms become favorable when the autonomous nervous system gradually achieves equilibrium. However, it was the detail of the operational method with retaining as many nerve fibers as possible that counts.

Vascular injury is always a concern in retroperitoneal NB surgery due to the tumor's proclivity for encasing visceral vessels. Massive hemorrhage, transfusion requirements, renal hypertension and unscheduled nephrectomy were all not uncommon following high-risk NB resection. A critical step for avoiding and minimizing injury to these vessels is their identification before they pass through the tumor, most often at their take-off from the aorta or vena cava [17]. Additionally, 3D-printed models could be of great assistance to pediatric surgeons in understanding the spatial relationships of tumors with adjacent anatomic structures, especially vessels [18]. Do not panic if vascular injury occurred during the operation. Perfusion can be sustained in most cases with primary suture and vascular anastomosis or prosthetic vascular graft repair.

Catecholamines released by the NB were a significant cause of preoperative hypertension, while postoperative hypertension was most likely renovascular. If postoperative hypertension is not actively treated, it might lead to cardiovascular or cerebrovascular problems and interfere with the effectiveness of chemotherapy. The majority of individuals could return to normal with hypotensors. Percutaneous transluminal angioplasty was considered for refractory hypertension that failed antihypertensive medications [19], and nephrectomy was often employed as a last resort.

Unscheduled organ excision primarily involved the kidney. Tumor invasion of the kidney and wrapping around the kidney or renal pedicle did not mean the need for nephrectomy, and when these occurred in our cohort, the kidney could be saved while the tumor was removed. The most frequent cause for organ removal was accidental blood vessel damage, which should be removed when there was postoperative organ atrophy or refractory symptoms such as resistant hypertension.

In 2009, the INRG created a new staging system that relies on preoperative imaging for staging [20, 21]. Central to the INRGSS are IDRFs combined with clinical data to provide upfront risk stratification. IDRFs are a consensus of radiologic findings across multiple organ systems that can be applied consistently to diagnostic imaging to describe organ, nerve, and vessel involvement [22]. Evidently, there was a direct correlation between IDRFs and complications, and complications were more likely to occur when more IDRFs were involved [10]. The presence of IDRFs as a risk factor for perioperative complications requires surgeons to pay more attention to patients with IDRFs during surgery, and this should not discourage the determination to pursue total resection of NB.

MYCN amplification is one of the strongest independent adverse prognostic factors, accounting for 20% to 25% of NB and is strongly associated with advanced-stage disease [23–25]. MYCN-amplified NB was sensitive to chemotherapy and could considerably lower IDRFs after chemotherapy. In patients with localized NB harboring MYCN amplification, extended surgery of the primary tumor site improved the local control rate and survival [12].

We observed that MYCN-amplified NB was extremely invasive to the vascular adventitia and adjacent tissues, fusing tumors, vessels, and surrounding tissue into a sticky mass, making the surgery more challenging and potentially increasing the occurrence of complications. Unexpectedly, MYCN amplification was not a significant risk factor for perioperative complications in the study, which should be due to the high incidence of LL and diarrhea. It was recommended that GTR should be carried out on MYCN-amplified NB, and suspicious adjacent tissue should be excised as much as possible, which may prevent local recurrence and improve prognosis to some extent.

Reports showed that operative complications had no significant adverse effect on EFS or OS [3], but this was not the case in this study. It is important to consider that the complication rate was associated with a high stage of INRGSS and risk group of INRG. In addition, any perioperative complication would result in delays in the timely delivery of systemic therapy and/or the ability to deliver full-dose therapies.

In conclusion, despite the high incidence of perioperative complications, the associated mortality rate was quite low. The presence of IDRFs, high-stage INRGSS, retroperitoneal lymph node metastasis, superior mesenteric artery encasement, and inferior mesenteric artery encasement were significant risk factors for perioperative complications of retroperitoneal NB. Surgery for retroperitoneal NB has always been a challenge for pediatric oncologists. To improve clinical outcomes for these patients, surgeons take on the challenge of eliminating these tumors. Future large-sample and multicenter studies are expected to explore additional information, develop a more intuitive understanding of the complications and risk factors for NB surgery, and assist surgeons in providing better management for NB patients.

## Data Availability

The datasets generated during and/or analyzed during the current study are available from the corresponding author on reasonable request.
